# Recurrence- and Malignant Progression-Associated Biomarkers in Low-Grade Gliomas and Their Roles in Immunotherapy

**DOI:** 10.3389/fimmu.2022.899710

**Published:** 2022-05-23

**Authors:** Chubei Teng, Yongwei Zhu, Yueshuo Li, Luohuan Dai, Zhouyang Pan, Siyi Wanggou, Xuejun Li

**Affiliations:** ^1^ Department of Neurosurgery, Xiangya Hospital, Central South University, Changsha, China; ^2^ Hunan International Scientific and Technological Cooperation Base of Brain Tumor Research, Xiangya Hospital, Central South University, Changsha, China; ^3^ Department of Neurosurgery, The First Affiliated Hospital, University of South China, Hengyang, China

**Keywords:** low-grade glioma (LGG), recurrence, malignant progression, cell cycle checkpoint, immune microenvironment, nomogram

## Abstract

Despite a generally better prognosis than high-grade glioma (HGG), recurrence and malignant progression are the main causes for the poor prognosis and difficulties in the treatment of low-grade glioma (LGG). It is of great importance to learn about the risk factors and underlying mechanisms of LGG recurrence and progression. In this study, the transcriptome characteristics of four groups, namely, normal brain tissue and recurrent LGG (rLGG), normal brain tissue and secondary glioblastoma (sGBM), primary LGG (pLGG) and rLGG, and pLGG and sGBM, were compared using Chinese Glioma Genome Atlas (CGGA) and Genotype-Tissue Expression Project (GTEx) databases. In this study, 296 downregulated and 396 upregulated differentially expressed genes (DEGs) with high consensus were screened out. Univariate Cox regression analysis of data from The Cancer Genome Atlas (TCGA) yielded 86 prognostically relevant DEGs; a prognostic prediction model based on five key genes (HOXA1, KIF18A, FAM133A, HGF, and MN1) was established using the least absolute shrinkage and selection operator (LASSO) regression dimensionality reduction and multivariate Cox regression analysis. LGG was divided into high- and low-risk groups using this prediction model. Gene Set Enrichment Analysis (GSEA) revealed that signaling pathway differences in the high- and low-risk groups were mainly seen in tumor immune regulation and DNA damage-related cell cycle checkpoints. Furthermore, the infiltration of immune cells in the high- and low-risk groups was analyzed, which indicated a stronger infiltration of immune cells in the high-risk group than that in the low-risk group, suggesting that an immune microenvironment more conducive to tumor growth emerged due to the interaction between tumor and immune cells. The tumor mutational burden and tumor methylation burden in the high- and low-risk groups were also analyzed, which indicated higher gene mutation burden and lower DNA methylation level in the high-risk group, suggesting that with the accumulation of genomic mutations and epigenetic changes, tumor cells continued to evolve and led to the progression of LGG to HGG. Finally, the value of potential therapeutic targets for the five key genes was analyzed, and findings demonstrated that KIF18A was the gene most likely to be a potential therapeutic target. In conclusion, the prediction model based on these five key genes can better identify the high- and low-risk groups of LGG and lay a solid foundation for evaluating the risk of LGG recurrence and malignant progression.

## Introduction

Low-grade gliomas (LGGs) make up about 7.6% of all brain tumors and 31.8% of gliomas. Occurring at all ages, LGGs have an incidence rate of 2.31/100,000 in the 0–14 years age group, 1.43/10,000 for 15–39 years of age, and 1.57/100,000 in the age group of 40 years and older. Besides, LGG incidence is higher in men (5.51/10,000) than in women (3.65/100,000) (1). Surgical resection remains the mainstay of treatment for LGGs, and adjuvant treatment with chemoradiotherapy is administered if needed. Reportedly, LGG patients have a 5-year survival rate of 70%–97% and a 10-year survival rate of 49%–76% (2, 3). About 52%–62% of patients have a recurrence within 5 years (4–6). In these recurrent cases, some have LGGs, while 17%–32% progress to high-grade gliomas (HGGs) (7–10). Worse prognosis of recurrent LGGs is predominantly a resultant of their malignant transformation. It is currently believed that the transition of LGGs to more aggressive HGGs is induced by the intrinsic diversity and heterogeneity of tumor cells that evolve to overt malignancy and develop resistance to therapy during tumor growth, eventually leading to a worse prognosis (11).

In the studies on the recurrence and evolution of gliomas by (12, 13) exome sequencing and DNA methylation data were derived from 23 and 19 patients before and after recurrence, respectively, for comparative analysis, from which the researchers discovered intratumoral heterogeneity during initial growth and subclones sharing epigenetic [glioma CpG island methylator phenotype (G-CIMP)] alterations, TP53 and ATRX mutations, and copy number alterations (12, 13). Although LGGs may come back without malignant progression after subtotal resection, some distinct subclones may give rise to HGGs spontaneously or following the use of temozolomide (TMZ). Particularly, spontaneous evolution is associated with alterations in cell cycle genes caused by gene mutations and promoter hypomethylation. Treatment-associated progression to HGGs involves downregulation of RB gene expression, activation of AKT-Mammalian Target Of Rapamycin (AKT-mTOR) pathway, genetic defects in the DNA mismatch repair (MMR) pathway, and hypermethylation of O^6^-methylguanine-DNA methyltransferase (MGMT) (13, 14). By comparing the DNA and RNA sequencing data of LGG patients with those with recurrent LGGs evolving to secondary glioblastoma (sGBM), Jiang et al. discovered that sGBM was significantly enriched with TP53 mutation, somatic hypermutation, MET-exon-14 (METex14) skipping, PTPRZ1-MET (ZM) fusion, and MET amplification; from their point of view, METex14 suppresses MET degradation and activates the Signal Transducer and Activator of Transcription 3 (STAT3) signaling pathway to promote tumor growth and angiogenesis and result in a significantly worse prognosis (15). Therefore, mutations in key genes and accumulation of mutations are the driving forces for the evolution and malignant progression of LGG.

Ample evidence has demonstrated the immunosuppressive nature of gliomas in the antitumor immune response. Antigen presentation was limited by the elevated expression of immunosuppressive factors in glioma cells such as programmed cell death ligand 1 (PD-L1) and indolamine 2,3-dioxygenase (IDO) (16). Glioma-associated microglia and macrophages (GAMs) inhibit immune cell activity by producing interleukin-10 (IL-10), chemokine C-C ligand 20 (CCL20), CCL22, and prostaglandin E2.5 (17). Moreover, regulatory T cells (Tregs) mediate immunosuppressive activities through phagocytosis of cytotoxic T lymphocytes in the glioma microenvironment (18). These immunosuppressive activities may induce tumor immune escape, promote the evolution of tumor cells, and eventually lead to recurrence and malignant progression of gliomas.

As sequencing technology advances, numerous large-scale tumor sequencing databases have been established to support tumor research with a sufficiently large sample size and abundant gene data resources. Additionally, the emergence of single-cell sequencing helps form the basis for studying the genetic characteristics of tumor subclones and deepening our knowledge regarding the characteristics of immune cell infiltration and the immune microenvironment. In this study, The Cancer Genome Atlas (TCGA) and Chinese Glioma Genome Atlas (CGGA) databases were applied to establish a transcriptomics-based gene prediction model that helps assess the risk of recurrence and malignant progression of LGGs for accurate and efficient prognosis prediction. On this basis, immune cell infiltration and the immune microenvironment in LGGs at varying risks of recurrence and malignant progression were characterized to evaluate potential immunotherapies.

## Materials and Methods

### Data Source

A total of 530 patients’ mRNA expressions sequenced by Illumina HiSeq were downloaded from TCGA-LGG from UCSC Xena database (https://xenabrowser.net/datapages/), and corresponding clinical information was also downloaded. After filtering based on overall survival status (OS) and overall survival days (OS.time), there remained 465 samples without any Not Available (NA) values (19). The CGGA sequence datasets (CGGA.mRNAseq_325 and CGGA.mRNAseq_693) were collected from CGGA database (http://www.cgga.org.cn) (20). The mRNA expression of normal brain tissue was collected from Genotype-Tissue Expression Project (GTEx, https://www.gtexportal.org) (21). To remove batch effects between GTEx and CGGA_325 datasets, we used the ComBat function in sva package to integrate both datasets.

### Differential Expression Analysis

The limma package of R was used to compare differences in gene expression in two defined groups, and the genes with significance cutoff criteria p value <0.05 (adjusted) and absolute fold change more than 2 were identified as the differentially expressed genes (DEGs).

### Prognosis Analysis

Univariate Cox regression analysis was used to identify the relationship between single signature and prognosis of patients. A single signature can be the mRNA expression value of a gene or a clinical feature. Multivariate Cox regression analysis was used to identify the relationship between multiple signatures and prognosis of patients. Univariate Cox and multivariate Cox regression analyses were performed by the coxph function of survival package of R.

### Dimension Reduction and Risk Score Modeling

The least absolute shrinkage and selection operator (LASSO) method was used to further reduce the less important features. The multivariate Cox regression with a stepwise procedure was used to filter the redundant variables and formed the final risk score model to predict the prognosis of the patients. The Beta values in multivariate Cox analysis were retracted as the gene coefficient. The surv_cutpoint function in the Survminer package was used to determine the optimal cutting point of the risk score, so that the samples in the dataset could be divided into high- and low-risk groups. The calculation of risk score is show:

Risk score = ∑ coef (i)*log 2 (counts (i)+1), where i represents the corresponding gene.

### Bio Function Analysis

The Gene Ontology (GO) enrichment analysis was performed based on DEGs by clusterprofile package of R (22). Based on Gene Set Enrichment Analysis (GSEA) and Gene Set Variation Analysis (GSVA) methods, we collected three gene sets (1,615 REACTOME gene sets, 186 Kyoto Encyclopedia of Genes and Genomes (KEGG) gene sets, 50 hallmark gene sets) from the Molecular Signatures Database (MSigDB v7.5.1) to explore enrichment signaling pathways between clusters or groups (23).

### Immune-Related Analysis

The abundance of six immune cell types, B cell, CD4 T cell, CD8 T cell, neutrophil, macrophage, and dendritic cell, in the tumor microenvironment is estimated using TIMER based on mRNA expression (24). The two other tools, CIBERSORT and xCell, were also used to estimate the abundance of member cell types in a mixed-cell population (25, 26). Several immune infiltration-related signatures from the study by Mariathasan et al. (27) were used to analyze their association with risk score.

### Correlation Analysis of Risk Score With Tumor Mutational Burden and Tumor Methylation Burden

Tumor mutational burden (TMB) is broadly defined as the number of somatic mutations per megabase of interrogated genomic sequence. The somatic mutation file *.maf of TCGA-LGG was downloaded from the GDC Data Portal (https://portal.gdc.cancer.gov) to calculate the TMB values. TCGA-LGG DNA methylation (HumanMethylation450) profile was downloaded from the UCSC Xena database (https://xenabrowser.net/datapages). Probes with a beta value greater than 0.8 were identified as sites of complete methylation, and the number of complete methylated probes was used to evaluate the tumor methylation burden. We used the abbreviations TMB.mut and TMB.met for tumor mutational burden and tumor methylation burden, respectively. The Pearson correlation was calculated by cor.test package of R.

### Nomogram Analysis of Risk Score With Clinicopathological and Molecular Characteristics

Univariate and multivariate Cox analyses were used to assess the independent prognostic factors of risk score compared with clinical features. Then, we used a nomogram to integrate risk score and clinical features to improve prognosis prediction accuracy. The performance of the nomogram model was tested using receiver operating characteristic (ROC) and decision curve analysis (DCA) curves of 1, 3, and 5 years.

### Cancer Cell Line Encyclopedia

Chronos, an algorithm for inferring gene knockout fitness effects based on an explicit model of cell proliferation dynamics after CRISPR gene knockout, was used to evaluate the effect of genes (28).

### Cell Culturing and Processing

The two glioma cell lines used in this study were U87 and U251, both from Xiangya Hospital. The cells were cultured in Dulbecco's Modified Eagle Medium (DMEM) containing 10% bovine serum in a 37°C, 5% CO_2_ incubator. Cells were inoculated on a six-well plate, and the experimental group was treated with BTB-1, a specific and reversible inhibitor of KIF18A. BTB-1 was dissolved with dimethylsulfoxide (DMSO) and diluted to a working concentration of 30 μM/L. Cells were treated for 24 h, and the control group was added with the same amount of DMSO (29). Finally, the two glioma cell lines were divided into four groups (U87 with DMSO, U87 with BTB-1, U251 with DMSO, and U251 with BTB-1).

### Evaluation of the Effect of BTB-1 on the Transcription Level of KIF18A in Glioma Cells

We used QRT-PCR to detect KIF18A mRNA levels in the four glioma cell lines, adding RNeasy Mini Kit (Qiagen, Hilden, NRW, Germany) to each group to extract total RNA. RNA was reversely transcribed into cDNA using Uni RT&qPCR Kit (TransGen Biotech, Beijing, China). Quantitative polymerase chain reaction was performed on Applied Biosystems QuantStudio 1 real-time fluorescent quantitative PCR system (Thermo Fisher Scientific, Waltham, MA, USA). Cycle conditions for qPCR were as follows: an initial denaturation step at 95°C for 15 min, followed by 40 cycles of amplification at 95°C, and annealing/extension at 56°C for 32 s. glyceraldehyde-phosphate dehydrogenase (GAPDH) was used as the internal control. Each experiment was carried out in triplicate. The primers were synthesized by Sangon Biotech, Shanghai, China. Their sequences are as follows: KIF18A, forward 5′-AAAAAGTGGTAGTTTGGGCTGA-3′ and reverse 5′-CTTTCAAGGGAGATGGCATTAG-3′; GAPDH, forward 5′-CATGAGAAGTATGACAACAGCCT-3′ and reverse 5′-AGTCCTTCCACGATACCAAAG-3′.

### Evaluation of the Effect of BTB-1 on Glioma Cell Proliferation

Cell Counting Kit-8 (CCK-8) was used to detect cell proliferation ability. Four groups of glioma cells were inoculated into 96-well plates with 5 wells in each group, and about 3,000 cells in each well were cultured in 100-μl medium for 0, 12, 24, and 36 h, respectively. Here, 10 μl CCK-8 solution was added to each well and incubated for 1.5 h. Absorbance was detected at 450-nm wavelength using absorbance Microplate reader (Spectra Max, ABS Plus), and Optical Density (OD) value was measured. The average OD values of each group were taken.

### Evaluation of the Effect of BTB-1 on Glioma Cell Cycle

Flow cytometry was used to detect cell cycle distribution to evaluate the effect of BTB-1 on glioma cell cycle. U87 and U251 cells with different processes were collected and washed twice with Phosphate Buffer Saline (PBS), then fixed with 70% ethanol at 4°C for 12 h, and then incubated with Prodium Iodide (PI)/RNase buffer. Flow cytometry (CYTEK Athena, USA) used red fluorescence and scattered light at 488 nm. The percentages of each cell cycle were analyzed.

### Gene Expression Data With Immunotherapy

For further immunotherapy research, the IMvigor210 dataset was downloaded to evaluate the predictive power of the risk score for immunotherapy [programmed cell death protein-1 (PD-1)] response, and it was available from http://research-pub.gene.com/IMvigor210CoreBiologies with completed information about the response to PD-L1 blockade (30).

### Statistical Analysis

The difference between two groups was compared by Wilcoxon test. Kaplan–Meier analysis with log-rank tests was used to perform survival curves. The ROC curve and corresponding Area Under the Curve (AUC) were generated by using the R package “timeROC.” * p-value <0.05, ** p-value <0.01, *** p-value <0.001, and p-value <0.05 are statistically significant. All analyses were performed using R 4.1.0 (https://cran.r-project.org).

## Results

### Identification of Recurrence Factors in Low-Grade Glioma

**Figure 1:**
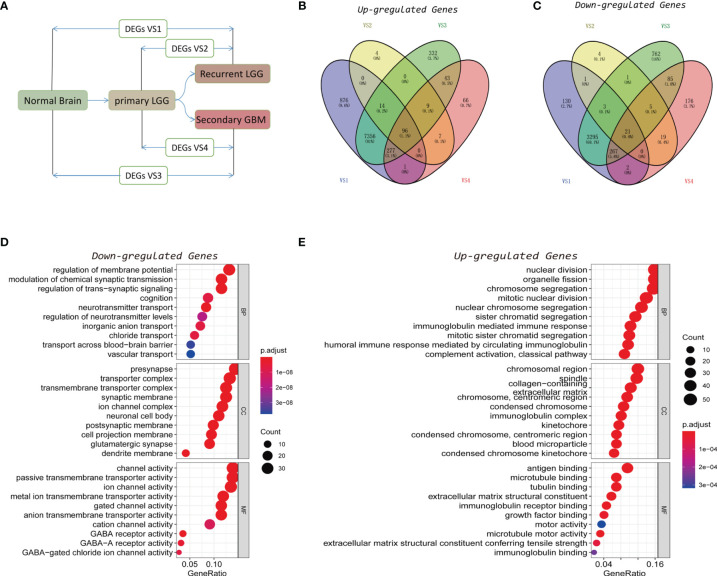
Identification of recurrence factors in low-grade gliomas. **(A)** Analysis design diagram of differentially expressed genes (DEGs). **(B, C)** Venn diagram was used to display the upregulated **(B)** and downregulated **(C)** genes in the four sets of DEGs. **(D, E)** Gene Ontology (GO) analysis of the consistent upregulated **(D)** and downregulated **(E)** genes in the four sets of DEGs.

Based on data acquired from CGGA and GTEx databases, a comprehensive analysis was carried out on the DEGs between normal brain tissue and recurrent LGG (rLGG), normal brain tissue and secondary GBM (sGBM), primary LGG (pLGG) and rLGG, as well as pLGG and sGBM (**Figure 1A**). A total of 4 DEG datasets were obtained, and DEGs with high consensus were defined as those with consistent differential expression trends in at least three datasets (**Supplementary Table S1**), and a Venn diagram was established to present the DEG datasets with upregulated and downregulated expressions (**Figures 1B, C** **)**. Eventually, 296 downregulated and 396 upregulated DEGs were screened through the above processes. Gene Ontology (GO) analysis was then used to annotate the functions of downregulated and upregulated DEGs. The biological process (BP) category of downregulated DEGs was mainly enriched in the regulation of membrane potential, modulation of chemical synaptic transmission, regulation of trans-synaptic signaling, etc. While for the cellular component (CC) category, the downregulated DEGs were associated with presynapse, transporter complex, transmembrane transporter complex, etc. In the molecular function (MF) category, the downregulated DEGs primarily participated in channel activity, passive transmembrane transporter activity, ion channel activity, etc. (**Figure 1D**). While the BP category of the upregulated DEGs was enriched mostly in the nuclear division, organelle fission, chromosome segregation, etc. In the CC category, the upregulated DEGs mainly showed associations with the chromosomal region, spindle, collagen-containing, etc. As for the MF category, the upregulated DEGs were involved primarily in antigen binding, microtubule binding, tubulin binding, etc. (**Figure 1E**).

### Identification of Main Contributors of Recurrence and Malignant Progression Factors and Construction of a Gene Signature Model

**Figure 2:**
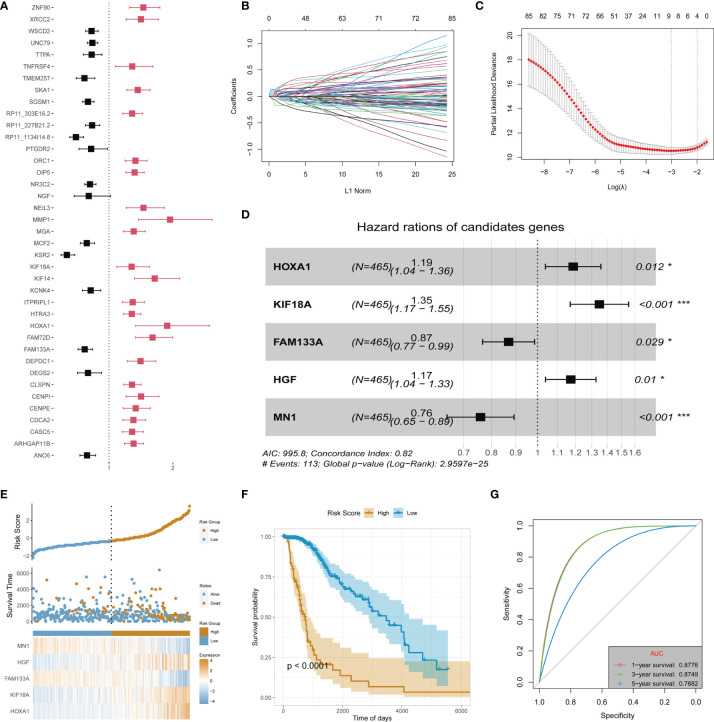
Identification of main contributors of recurrence and malignant progression factors and construction of risk signature in TCGA-LGG cohort. **(A)** Univariate Cox analysis of all recurrence factors. **(B)** Cross-validation for tuning the coefficient selection in the least absolute shrinkage and selection operator (LASSO) regression. **(C)** LASSO regression of the 86 OS-associated genes. **(D)** Multivariate Cox regression analysis for the 5 genes. **(E)** Allocation of patients in the training set on the basis of the risk score. **(F)** Kaplan–Meier curves display the diversity in OS between the high-risk and low-risk groups in the training set. **(G)** Area Under the Curve (AUC) of time-dependent receiver operating characteristic (ROC) curves examined the prognostic performance of the risk score in the training set. "*" means 0.01<p<0.05, "***"means p<0.001.

Recurrence and malignant progression of LGG constitute the primary factors affecting its prognosis. Therefore, in order to determine the main contribution factors, the data of LGG patients were extracted from TCGA database for univariate Cox regression analysis on the identified highly consistent DEGs. The False Discovery Rate (FDR) values of the training set and test set are used for filtering. It was found that 24 downregulated and 62 upregulated DEGs were associated with the prognosis of LGG (p < 0.05) (**Supplementary Table S2**). The top 39 DEGs with statistically significant differences are shown in **Figure 2A**. Subsequently, LASSO regression, one of the techniques in machine learning, was adopted to dispose of dimension reduction of the 86 DEGs with prognostic significance (**Figures 2B, C** **)**. Multivariate Cox regression was then used to establish the prognosis prediction model to identify the main contributors. Corresponding results revealed that HOXA1, KIF18A, FAM133A, hepatocyte growth factor (HGF), and MN1 were the primary contributors (p < 0.05) (**Figure 2D**). Finally, the patients in the training dataset were stratified according to the risk scores obtained in the 5-gene signature model (**Figure 2E**). It was found that the established model could well predict the prognosis of TCGA-LGG patients, showing good stability at the same time (Area Under the Curve (AUC) of 1-, 3-, and 5-year ROC: 0.8776, 0.8749, and 0.7682, respectively) (**Figures 2F, G**).

### Validation of Risk Signature in the Test Dataset and Validation Dataset

**Figure 3:**
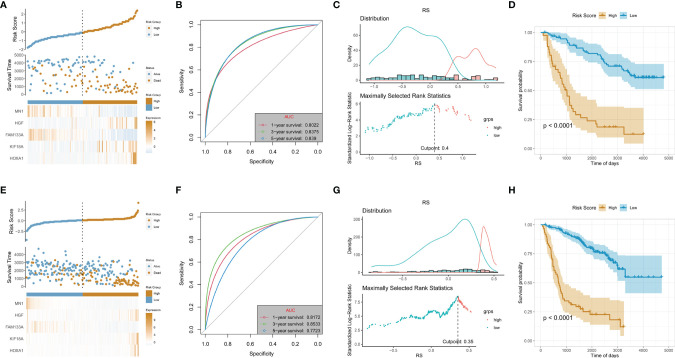
Validation of risk signature in the test dataset (CGGA_325 cohort) and validation dataset (CGGA_693). **(A, E)** Allocation of patients in the training set on the basis of the risk score in the test and validation dataset. **(B, F)** Area Under the Curve (AUC) of 1-, 3-, and 5-year receiver operating characteristic (ROC) curves examined the prognostic performance of the risk score in the test and validation dataset. **(C, G)** Determine the optimal cut point for risk score in the test and validation dataset. **(D, H)** Kaplan–Meier curves display the diversity in OS between the low- and high-risk score groups in the test and validation dataset.

Two sub-datasets of CGGA (CGGA325 and CGGA693) were collected to verify the effectiveness and stability of the 5-gene signature prediction model. In the CGGA325 dataset, the model could well stratify the high-risk and low-risk LGG groups. Survival analysis showed that the prognosis of the high-risk group was significantly worse than that of the low-risk group (p < 0.05) (**Figures 3A–D**). Furthermore, in the CGGA693 dataset, the model could still better stratify the risk of LGG patients, and the prognosis of the high-risk group was also obviously worse than that of the low-risk group, as indicated by survival analysis (p < 0.05) (**Figures 3E–H**).

### The Gene Set Enrichment Analysis of the High- and Low-Risk Groups

**Figure 4:**
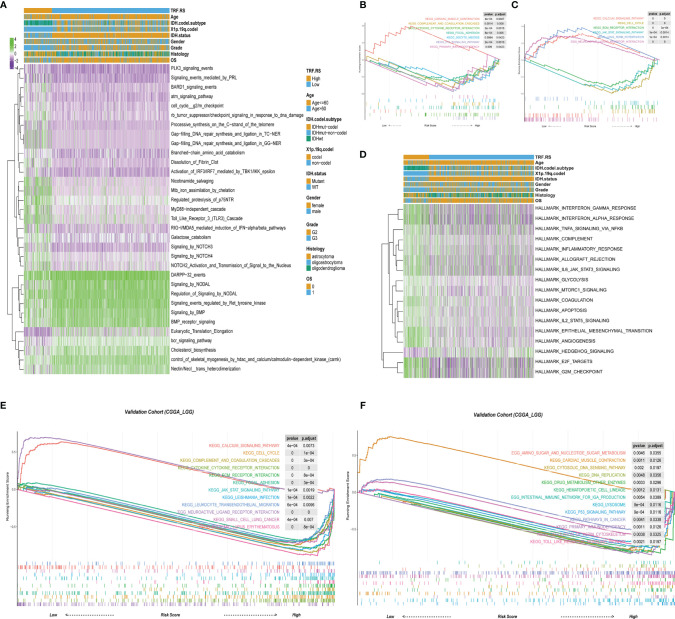
The biology function enrichment analysis between the low- and high-risk score groups. **(A)** Gene Set Variation Analysis (GSVA) results based on the REACTOME gene sets. **(B, C)** Gene Set Enrichment Analysis (GSEA) results based on the Kyoto Encyclopedia of Genes and Genomes (KEGG) gene sets. **(D)** GSVA results based on the hallmark gene sets. **(E, F)** GSEA results based on the KEGG gene sets in the validation cohort (CGGA-LGG).

In order to clarify the mechanism of LGG recurrence or malignant progression, the differences in signaling pathways between high-risk and low-risk groups were analyzed based on TCGA-LGG dataset. Meanwhile, GSVA and GSEA were performed to analyze the signaling pathway differences between high-risk and low-risk groups based on REACTOME gene set, KEGG gene set, and hallmark gene set. In the REACTOME gene set, several signaling pathways were significantly activated in the high-risk group compared with the low-risk group, including cell cycle G2/M checkpoint, RB tumor suppressor/checkpoint signaling in response to DNA damage, Gap-filling DNA repair synthesis and ligation in TC-NER, Toll-like receptor 3 (TLR3) cascade (**Figure 4A**). KEGG gene set-based analysis revealed that signaling pathways such as cell cycle, extracellular matrix (ECM)–receptor interaction, Janus kinase/signal transducers and activators of transcription (JAK-STAT) signaling pathway, complement and coagulation cascades, and cytokine receptor interaction were obviously activated in the high-risk group, while calcium signaling pathway, long-term potentiation, neuroactive ligand–receptor interaction, etc., were inactivated in the high-risk group (**Figures 4B, C** **)**. At the same time, similar results can be obtained by the CGGA database based on KEGG GSEA (**Figures 4E, F** **)**. In the tumor-specific gene set of hallmark, there existed activation in cell cycle G2/M checkpoint and tumor immune-related signaling pathways (**Figure 4D**). Collectively, these signals are mainly related to tumor immune regulation and DNA damage-related cell cycle checkpoints, indicating that these mechanisms may be significantly involved in LGG recurrence and malignant progression.

### Infiltrated Immune Cells in High- and Low-Risk Score Groups From the Training and Validation Cohorts

**Figure 5:**
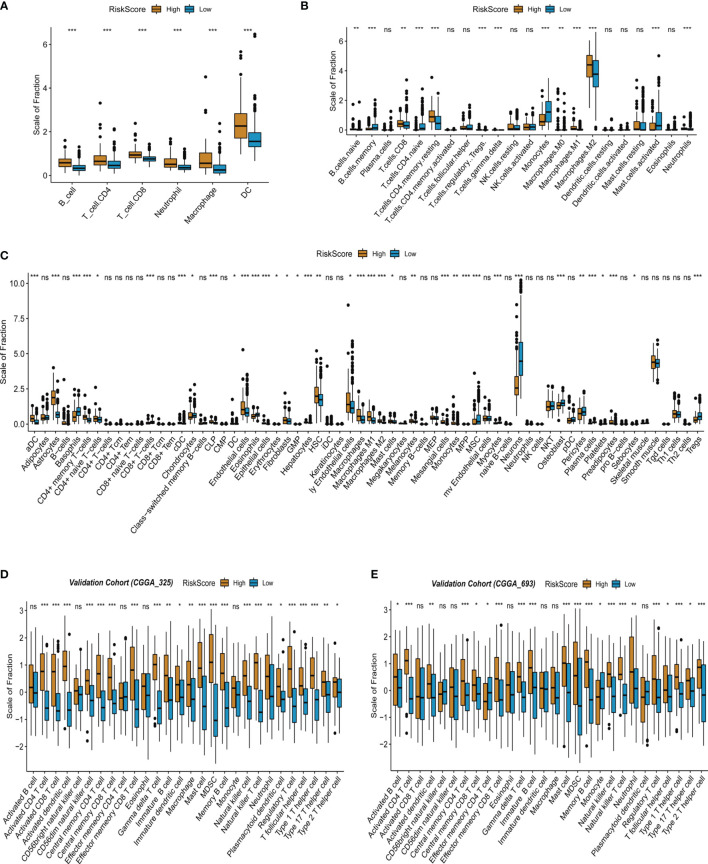
Infiltrated immune cells in the high- and low-risk score group from the training and validation cohorts. **(A)** Immune infiltrates in the two groups based on the TIMER algorithm from TCGA-LGG cohort. **(B)** Immune infiltrates in the two groups based on the CIBERSORT algorithm from TCGA-LGG cohort. **(C)** Immune infiltrates in the two groups based on the xCell algorithm from TCGA-LGG cohort. **(D)** Immune infiltrates in the two groups based on the ssGSEA algorithm from the validation cohort (CGGA 325). **(E)** Immune infiltrates in the two groups based on the ssGSEA algorithm from the validation cohort (CGGA 693). "*" means 0.01<p<0.05, "**"means 0.001<p<0.01, "***"means p<0.001, "ns"means no significance.

Based on the above exploration, multiple tumor immune-related pathways were discovered to be activated in the high-risk group of TCGA-LGG, suggesting an intimate association of the tumor immune microenvironment with LGG recurrence and malignant progression. Accordingly, the infiltration of immune cells was further evaluated in the high- and low-risk score groups of TCGA-LGG. The abundance of immune cells in the tumor microenvironment was evaluated by the TIMER algorithm according to mRNA expressions. The results showed that the infiltration of B cells, CD4 T cells, CD8 T cells, neutrophils, macrophages, and dendritic cells in the high-risk score group was stronger than that in the low-risk score group of LGG (**Figure 5A**). Further analyses were continued by using CIBERSORT and the xCell algorithm. Analysis based on CIBERSORT revealed that the infiltration of anti-inflammatory immune cells such as CD8 T cells, CD4 memory resting T cells, and M2 macrophages was stronger in the high-risk score group than that in the low-risk score group (**Figure 5B**). xCell-based analysis also indicated that the infiltration of most immune cells increased in the high-risk score group (**Figure 5C**). Collectively, these data suggest that the high-risk score group of LGG has stronger immune cell infiltration than that of the low-risk score group. In addition, using CGGA database (CGGA325 and CGGA693) as validation sets, we also found that immune cell infiltration in the LGG high-risk group was stronger than that in the low-risk group by ssGSEA algorithm (**Figures 5D, E** **)**.

### Genomic and Methylation Characteristics in the High- and Low-Risk Score Groups From TCGA-LGG Cohort

**Figure 6:**
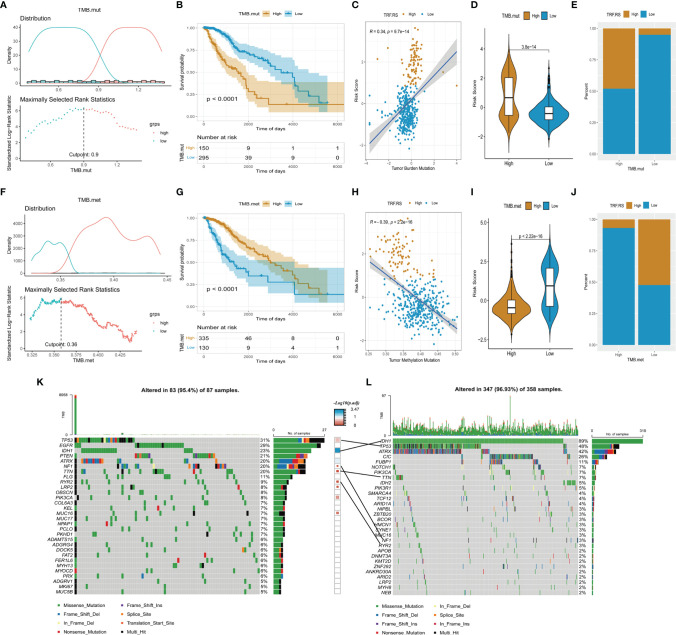
Genomic and methylation characteristics in the high- and low-risk score group from TCGA-LGG cohort. **(A, F)** Determine the optimal cut point for TMB.mut and TMB.met. **(B, G)** Comparison of overall survival between the low and high group of TMB.mut and TMB.met. **(C, H)** Scatter distribution of the risk score with TMB.mut and TMB.met. **(D, I)** Comparison of the risk score between the low and high group of TMB.mut and TMB.met. **(E, J)** Comparison of the proportion of risk score type between the low and high group of TMB.mut and TMB.met. **(K, L)** Oncoplot of the high and low group of risk score.

As mentioned above, DNA damage-related cell cycle checkpoint pathways were activated in the high-risk score group of LGG, suggesting that there may be more frequent gene mutations in this group. For an in-depth understanding of gene mutations in the high- and low-risk score groups of LGG, further grouping was performed based on TMB (TMB.mut), including high and low TMB.mut groups of LGG (**Figure 6A**). Survival analysis between high and low TMB.mut groups showed that the prognosis of high TMB.mut was significantly poorer than that in the low TMB.mut group (p < 0.001) (**Figure 6B**). Correlation analysis revealed a good positive correlation between risk factor stratification and TMB.mut stratification (R = 0.34, p < 0.05), that was, the mutation burden of the high-risk score group was significantly higher than that of the low-risk score group of LGG (**Figures 6C-E**). Simultaneous exploration was performed on the level of methylation in the high- and low-risk score groups of LGG. Similarly, two groups of high and low TMB.met were divided based on tumor methylation burden (TMB.met) (**Figure 6F**). Survival analysis indicated a significantly poorer prognosis in the low TMB.met group than that in the high TMB.met group (p < 0.001) (**Figure 6G**); moreover, the correlation analysis presented a negative correlation between risk factor stratification and TMB.met stratification (R = -0.39, p < 0.05), suggesting that the DNA methylation level of the LGG high-risk score group was remarkably lower than that of the low-risk score group (**Figures 6H–J**). We further analyzed the mutation of major driver genes in the high- and low-risk groups, and the driver genes with a high mutation frequency in the high-risk group were TP53, EGFR, IDH1, etc. (**Figure 6K**). The driver genes with a high mutation frequency in the low-risk group were IDH1, TP53, ATRX, etc. (**Figure 6L**). TP53 mutation had a strong coexisting relationship with the high-risk group, and IDH1 mutation had a strong coexisting relationship with the low-risk group (**Figure 6L**).

### Independent Prognostic Value of the Tumor Recurrence Factor (TRF) Risk Score

**Figure 7:**
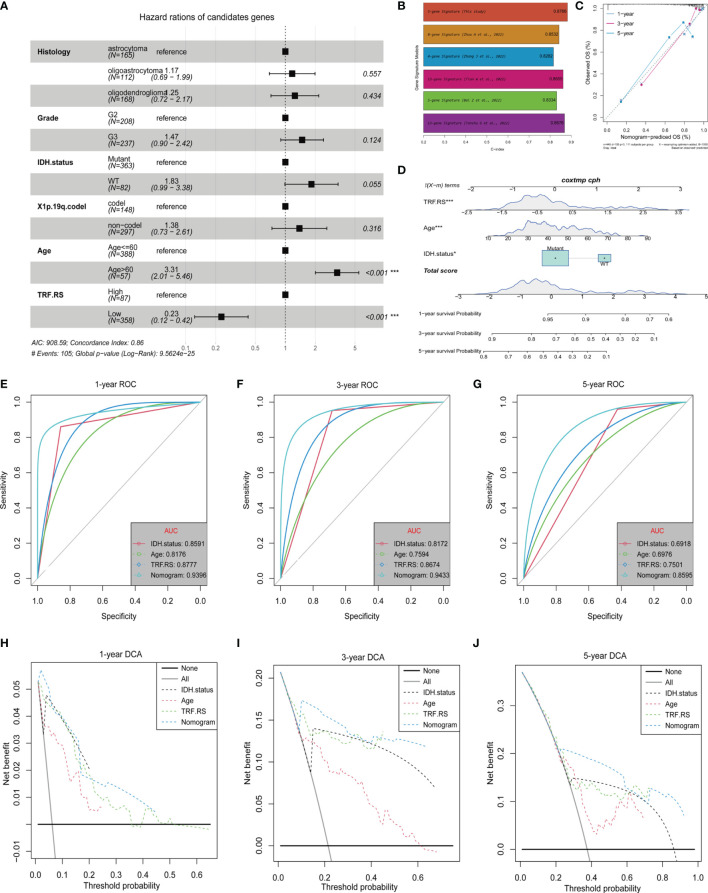
Independent prognostic value of the Tumor Recurrence Factor risk score. **(A)** Forest plot of multivariate Cox analysis between the Tumor Recurrence Factor risk score and clinical traits. **(B)** Comparison of the prediction power between this study and other studies. **(C)** Calibration plot was used to predict the 1-, 3-, and 5-year survival in TCGA-LGG cohorts. **(D)** Nomogram of TCGA-LGG cohorts was used to predict overall survival. **(E–G)** Receiver operating characteristic (ROC) curves showed the 1-, 3-, and 5-year predictive efficiency of risk score, age, IDH mutation status, and nomogram model. **(H–J)** Decision curve analysis (DCA) curves showed the 1-, 3-, and 5-year predictive efficiency of risk score, age, IDH mutation status, and nomogram model. "*" means 0.01<p<0.05, "***"means p<0.001.

Subsequent analyses were conducted to integrate the 5-gene signature prediction model established based on its expression characteristics with other clinical features and promote its application in prognosis judgment. Firstly, multivariate Cox regression analysis was performed for determining the independent prognostic factors of Histology, Grade, IDH.status, X1p.19q.codel, Age, and Tumor Recurrence Factor (TRF) risk score (**Figure 7A**). The Tumor Recurrence Factor risk score and Age were found to be relatively significant independent prognostic factors (p < 0.001), followed by IDH.status (p = 0.055) (**Figure 7D**). After that, a nomogram was constructed based on IDH.status, Age, and Tumor Recurrence Factor risk score to predict the prognosis of TCGA-LGG patients. Using the Calibration diagram to verify the prediction efficacy of the constructed nomogram, it could be found that its 1-, 3-, and 5-year prognostic prediction efficacy had high accuracy and stability (**Figure 7C**). Furthermore, the ROC curve was then drawn to compare the prediction efficacy of IDH.status, Age, Tumor Recurrence Factor risk score, and nomogram model. It could be noticed that the 1-, 3-, and 5-year prognostic prediction efficacy of the nomogram was higher than that of other features in 1, 3, and 5 years. Among them, the Area Under the Curve (AUC) of 1-year ROC was 0.8591 (IDH.status), 0.8176 (Age), 0.8777 (Tumor Recurrence Factor risk score), and 0.9396 (nomogram) (**Figure 7E**); that of 3-year ROC was 0.8172 (IDH.status), 0.7594 (Age), 0.0.8674 (Tumor Recurrence Factor risk score), and 0.9433 (nomogram) (**Figure 7F**); and that of 5-year ROC was 0.6918 (IDH.status), 0.6976 (Age), 0.7501 (Tumor Recurrence Factor risk score), and 0.8595 (nomogram) (**Figure 7G**). Furthermore, the DCA curve was generated to examine the prediction efficacy of IDH.status, Age, Tumor Recurrence Factor risk score, and nomogram model. Similar results were observed as described above, which support the strong stability of the prediction efficacy of the constructed nomogram (**Figures 7H–J**). Moreover, we selected some relevant literature to compare the predictive power of the prognostic models. Compared with other models, our prediction model has better predictive power (**Figure 7B**) (31–35).

### Evaluation of Potential Therapeutic Targets of Risk Signature Genes Based on the CCLE Database

**Figure 8:**
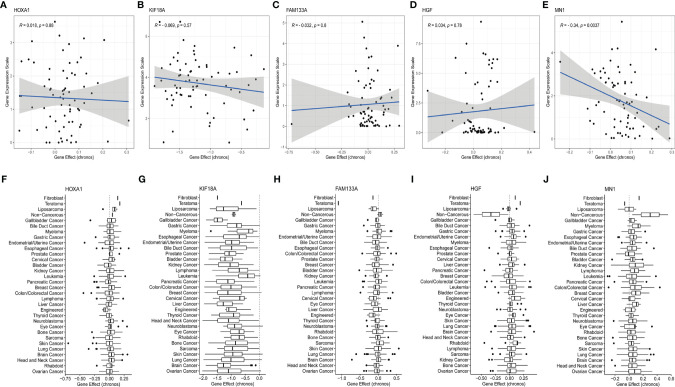
Evaluation of the potential therapeutic targets of risk signature genes based on the CCLE database. **(A–E)** Scatter distribution of gene expression and gene effect of the 5 genes, including HOXA1, KIF18A, FAM133A, HGF, and MN1, in brain cancer cell lines. **(F–J)** Distribution of gene effects of the 5 genes, including HOXA1, KIF18A, FAM133A, HGF, and MN1, in various cancer cell lines.

In order to further explore whether the genes of the main contributors were potential therapeutic targets, the responses of the five genes to different tumor cell lines after inhibition were evaluated based on the CCLE database. KIF18A and MN1 were discovered to be potential therapeutic targets. Specifically, KIF18A, as a risk factor, could significantly reduce the proliferation of most tumor cell lines (including brain tumor cell lines) (**Figures 8B, G** **)**, while MN1, as a protective factor, could enhance the proliferation of most tumor cell lines (including brain tumor cell lines) (**Figures 8E, J** **)**. However, the other 3 genes could not induce corresponding changes in tumor cell lines after being inhibited (**Figures 8A, C, D, F, H, I**), suggesting no potential therapeutic target value.

### Evaluation of the Effect of KIF18A Inhibitor BTB-1 on the Function of Glioma Cell Lines

**Figure 9:**
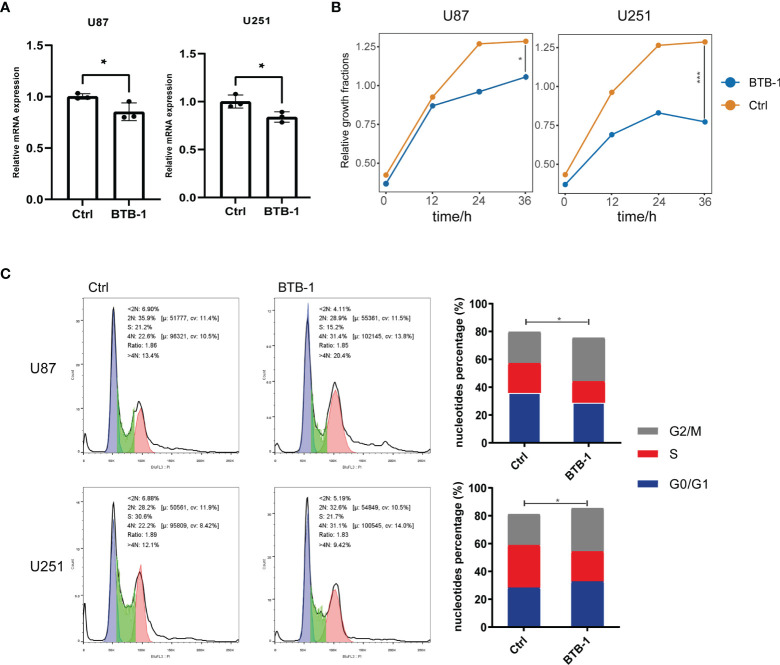
Evaluation of the effect of KIF18A inhibitor BTB-1 on the function of glioma cell lines. **(A)** Evaluation of the effect of BTB-1 on the transcription level of KIF18A in glioma cells. **(B)** Evaluation of the effect of BTB-1 on glioma cell proliferation. **(C)** Evaluation of the effect of BTB-1 on glioma cell cycle. "*" means 0.01<p<0.05, "***"means p<0.001.

As mentioned above, KIF18A was found to be a potential therapeutic target. Therefore, to evaluate its potential therapeutic value, we treated glioma cells with BTB-1, a specific small-molecule inhibitor of KIF18A, to evaluate its effect on glioma cell function. KIF18A mRNA expression in U87 and U251 cells decreased (p < 0.05) (**Figure 9A**). It suggested that BTB-1 could inhibit the expression of KIF18A. As a further study on the effect of BTB-1 on cell proliferation, CCK-8 test showed that in U87 and U251 cell lines, the cell proliferation rate of the BTB-1 treatment group was lower than that of the control group. It was suggested that BTB-1 could inhibit cell proliferation (**Figure 9B**). The results of flow cytometry were shown in **Figure 9C**. The G2/M phase ratios of U87 and U251 cells treated with BTB-1 were 31.4% and 31.1%, and those of the control group were 22.6% and 22.2%, respectively, with statistically significant differences (p < 0.05). In conclusion, the cell cycle of BTB-1-treated glioma cells stagnated in G2/M phase, and the proportion of cells in the G2/M phase increased. These results suggest that BTB-1 inhibits the proliferation of tumor cells mainly by inhibiting KIF18A and arresting glioma cells in the G2/M phase.

### Evaluation of Response to Immunotherapy Based on the Tumor Recurrence Factor Risk Score

**Figure 10:**
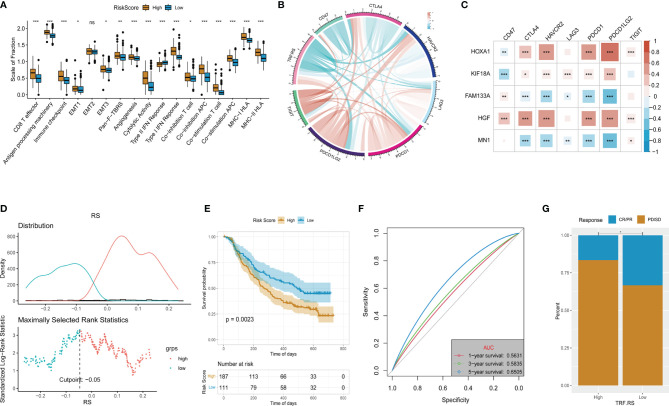
Evaluation of the response to immunotherapy. **(A)** Comparation of the immune response signature between the high- and low-risk groups. **(B)** Corelationships between the Tumor Recurrence Factor risk score and common immune checkpoints. **(C)** Corelationships between the 5 key genes and common immune checkpoints. **(D)** Determine the optimal cut point for the Tumor Recurrence Factor risk score. **(E)** Comparison of the overall survival between the low and high Tumor Recurrence Factor risk score. **(F)** Receiver operating characteristic (ROC) curves showed the 1-, 3-, and 5-year predictive efficiency of the Tumor Recurrence Factor risk score. **(G)** Comparison of the proportion of response to treatment between the low and high Tumor Recurrence Factor risk score. "*" means 0.01<p<0.05, "**"means 0.001<p<0.01, "***"means p<0.001,"ns"means no significance.

To test whether this predictive model can predict the immunotherapy response of LGG, we first assessed the differences in key factors in the immune response process between the high- and low-risk groups and found that the expression of these factors was higher in the high-risk group than that in the low-risk group (**Figure 10A**). Further analysis of the association between the Tumor Recurrence Factor risk score and common immune checkpoints of tumors showed that the Tumor Recurrence Factor risk score was correlated with CD47, CTLA7, HAVCR2, LAG3, PDCD1, and other immune checkpoints (**Figure 10B**). Independent analysis of the association between the 5 genes and immune checkpoints showed that all of the 5 genes were closely related to immune checkpoints (**Figure 10C**). Finally, the IMvigor210 dataset model (a database of bladder cancer) was selected and patients with bladder cancer in the database were divided into the high- and low-risk score groups (**Figure 10D**). Meanwhile, survival analysis showed that the model had a significant difference in prognosis between the high- and low-risk score groups of bladder cancer (**Figure 10E**), with relatively good stability in predicting the prognosis (**Figure 10F**). Analysis of the data of immunotherapy effect in the database revealed that the low-risk score group had a better response to immunotherapy than that in the high-risk score group (**Figure 10G**). With respect to the above, the constructed gene signature model not only has good universality and stability in predicting tumor prognosis but also provides a potential reference for predicting tumor response to immunotherapy.

## Discussion

LGG has a better prognosis than that of HGG, which, however, shows a deterioration in its prognosis primarily owing to the risk of recurrence and malignant progression, becoming a critical concern in the treatment of LGG. Based on transcriptome analysis, a new prediction model was established in our study, which can effectively evaluate the risk of LGG and predict the risk of recurrence and malignant progression. Finally, a total of 5 genes related to the recurrence and malignant progression of LGG were screened in our study, including three risk factors (HOXA1, HGF, and KIF18A) and two protective factors (FAM133A and MN1).

HOXA1 is highly expressed in breast cancer, ovarian cancer, gastric cancer, and various other cancers, which has regulatory roles on cell differentiation, apoptosis, and migration (36–38). For instance, in glioblastoma, long non-coding RNA (lncRNA) HOTAIRM1 has been documented to upregulate the expression of HOXA1 by blocking the binding of G9a and EZH2 to histones H3K9 and H3K27, resulting in the inability of methyltransferase to methylate the promoter of HOXA1 (39). At present, there is no relevant research on its downstream pathway in glioma. HOXA1 was found to upregulate the expression of BCL2 in breast cancer, which could also enhance cell proliferation and promote malignant transformation of cancer cells by activating STAT5a/b and MAPK signaling pathways (40). Furthermore, HGF is mainly produced by stromal cells, and its receptor is the mesenchymal–epithelial transition (MET) factor (41). HGF can bind to MET to activate various pathways such as Ras/MAPK, PI3K/Akt, and STAT, thereby regulating cell growth, movement, and morphogenesis (42–45). It has been reported that HGF and MET were highly expressed in glioma to promote tumor growth and angiogenesis, which could also boost tumor evolution and malignant progression to HGG by activating AKT and MAPK pathways (46, 47). Meanwhile, KIF18A belongs to the kinesin 9 family. It has a significantly upregulated expression in G2/M phase, which can contribute to the arrangement of chromosomes on the equatorial plate by adjusting the length of microtubules (Mts), ensuring the normal separation of sister chromosomes and normal progression of mitosis (48–51). High expression of KIF18A has been confirmed in multiple cancers such as breast cancer, lung cancer, and colorectal cancer (52–54). Prior cell function experiments showed that overexpression of KIF18A in the HeLa cell line could significantly increase the number of cells with multipolar mitotic spindles (50). At the same time, KIF18A can combine with microtubules (Mts), move along Mts, and has microtubule depolymerizing activity, both of which require the participation of ATP as a major substrate to supply energy (55, 56). Accordingly, considering the ATPase activity of KIF18A, Catarinella et al. (29) developed a small-molecule drug BTB-1, which can inhibit KIF18A in an ATP-competitive and Mt-uncompetitive manner; and tumor cells after BTB-1 treatment showed abnormal spindle formation and chromosome division, resulting in tumor cell apoptosis. In our study, analysis of data acquired from the CCLE database revealed the potential therapeutic target significance of KIF18A, that was, inhibiting its expression or function could inhibit tumor growth. Further *in vitro* experiments on glioma cell lines (U87 and U251) confirmed that KIF18A’s specific inhibitor BTB-1 inhibited the proliferation of glioma cells significantly, and the cell cycle stagnated in G2/M phase. Therefore, the mechanism of treating glioma with KIF18A as a target is worthy of further study.

FAM133A is a type of Cancer-Testis Antigen (CTA), a class of proteins that is highly expressed in immune privilege sites such as the brain and testis (57). High expression of FAM133A in cervical cancer and pancreatic cancer has been reported to be associated with the malignant degree of tumors (58, 59). While in glioma, FAM133A is the downstream target of miR-155. The low expression of miR-155 in IDH1-mutant glioma can upregulate the expression of FAM133A, which may further reduce the invasiveness and proliferation of IDH1-mutant glioma by targeting Matrix metalloproteinase-14 (MMP14) (60). Therefore, consistent with previous reports, this study indicated that LGG patients with a high expression of FAM133A had a better prognosis. Furthermore, MN1 was initially found to cause gene rearrangement by chromosomal balanced translocation, which plays an important role in the pathogenesis of meningioma and myeloproliferative diseases (61, 62). The loss-of-function mutation of MN1 may induce CEBALID syndrome, which is manifested as neurodevelopmental impairments and facial deformities (63). According to current research, the genomic changes of MN1 are predominated by rearrangement (64–67), with the report of the deletion and increase of adjacent loci as well (67). Moreover, in astroblastoma or neuroepithelial tumors, patients with MN1 rearrangement were reported to have longer survival and better prognosis (65, 68). However, some researchers considered no significant superiority in the clinical progression and prognosis of these patients (69). So far, it is unknown with respect to the relationship between the expression of MN1 at the transcriptome level and the prognosis of glioma. In the present study, the prognosis of LGG patients was poorer in the case of low expression of MN1, accompanied by a higher risk of progression to HGG. Further analysis of data acquired from the CCLE database revealed stronger cell proliferation and invasion abilities in cell lines of brain tumors with the silencing of MN1 expression. Consequently, it is believed that MN1 may be related to the normal development and differentiation of the nervous system. Its low expression may increase the stemness of glioma cells to boost the malignant progression of glioma. Its role and mechanism in the malignant progression of glioma need to be further studied.

Furthermore, in our study, the abnormality of DNA damage-related cell cycle checkpoints showed an intimate association with the recurrence and malignant progression of LGG. The cell cycle consists of four phases: G1, S, G2 and M. Cells have a strict cell cycle checkpoint mechanism to ensure the correct transmission of genetic materials to the next generation of cells. Dysfunction in checkpoints can lead to abnormal proliferation of cells and tumorigenesis (70). Under normal circumstances, in the case of DNA damage, the ATR-CHK1-CDC25 pathway may be activated to inhibit cyclinA/B-CDK1 complex and hence block the cell replication arresting at G2 phase (71). While the CDK1 gene is overexpressed in glioma, which may promote the G2 phase transition of cells with DNA damage. It has been documented that the expression of CDK1 is positively correlated with the grade of glioma, which may also increase with malignant progression after recurrence (72–74). In addition, Rb is a tumor suppressor, and its inactivation by mutation can activate E2F protein to overexpress cyclinE/A. The cyclinE/A-CDK2 is an important complex of cell cycle checkpoints. The overexpressed cyclinE/A plays a critical promoting role in G1/S cell transition of cells with DNA damage (75). Deletion or downregulation of Rb gene is common in gliomas (76), and its downregulation is more common in HGG (77, 78). Moreover, van Thuijl et al. (14) believed that patients with LGG treated with TMZ can induce Rb gene mutation, resulting in tumor recurrence and progression to HGG. Collectively, in our opinion, there may be a regulation disorder in the cell cycle checkpoints related to DNA damage through various mechanisms during the progression of LGG to induce continuous accumulation of genomic mutations, leading to tumor evolution and further progression to HGG.

Immune cell infiltration in the tumor, as an indicator of the tumor microenvironment, plays a critical role in the recurrence and progression of glioma. The proposed impact is double-edged generally. To be specific, while immune cells play a role in discouraging tumor progression, due to the “immune escape” mechanism, besides avoiding attack by the immune system, tumor cells can even “tame” immune cells by regulating the phenotype and function of immune cells (e.g., secreting cytokines, chemokines, etc.) and hence contribute to a microenvironment conducive to tumor progression (79–81). On the other hand, the “tamed” immune cells can also secrete cytokines to regulate the DNA methylation of tumor cells, thereby promoting the evolution of tumors (17). In this study, the degree of immune cell infiltration was significantly higher in the LGG high-risk group than that in the low-risk group. GSEA and GSVA also found that immunosuppressive and anti-inflammatory pathways were significantly activated in the high-risk group. It is suggested that LGG in the high-risk group can create an immunosuppressive microenvironment more conducive to tumor growth and evolution through tumor–immune cell interaction under stronger immune cell infiltration. Moreover, an immune barrier is developed in the tumor marginal microenvironment by immune cells at the periphery of tumors, which may facilitate the development of LGG chemoradiotherapy resistance and boost the recurrence and malignant progression of LGG (82).

## Conclusions

We obtained 5 genes (HOXA1, HGF, KIF18A, FAM133A, MN1) associated with LGG recurrence and malignant progression through differential expression gene analysis and established a stable and efficient prognostic model based on these. Among the five genes, KIF18A is considered to be the most significant potential therapeutic target. Its specific inhibitor BTB-1 can block the cell cycle of glioma cells in G2/M phase to inhibit the proliferation of glioma cells. We also found that abnormal DNA damage-related cell cycle checkpoints and changes in the tumor immune microenvironment may be important mechanisms leading to tumor cell evolution and LGG progression to HGG.

## Data Availability Statement

No new data were generated in this study, and all data used in this study are publicly available online. All the codes in this study are available on GitHub, linked to: https://github.com/Richard-Li-lab-team/LGG_TRF.

## Author Contributions

CT conceived and designed the experiments, authored and reviewed drafts of the paper, and approved the final draft. YZ analyzed the data, prepared figures and tables, and approved the final draft. YL helped to complete validation experiments. LD prepared figures and tables, collected literature, and approved the final draft. ZP collected literature and approved the final draft. SW conceived and designed the experiments, reviewed drafts of the paper, and approved the final draft. XL conceived and designed the experiments, reviewed drafts of the paper, and approved the final draft.

## Conflict of Interest

The authors declare that the research was conducted in the absence of any commercial or financial relationships that could be construed as a potential conflict of interest.

## Publisher’s Note

All claims expressed in this article are solely those of the authors and do not necessarily represent those of their affiliated organizations, or those of the publisher, the editors and the reviewers. Any product that may be evaluated in this article, or claim that may be made by its manufacturer, is not guaranteed or endorsed by the publisher.
